# Pathogenesis and distribution of infective endocarditis in the pediatric population: a 20-year experience in a tertiary care center in a developing country

**DOI:** 10.3389/fcvm.2023.1182468

**Published:** 2023-08-17

**Authors:** Moustafa Rashed, Ghina Fakhri, Rana Zareef, Nour Abdul Halim, Mohamed Ahmed, Ghassan Dbaibo, Issam El-Rassi, Fadi Bitar, Mariam Toufic Arabi

**Affiliations:** ^1^ Department of Pediatrics and Adolescent Medicine, American University of Beirut Medical Center, Beirut, Lebanon; ^2^ Department of Family Medicine, American University of Beirut Medical Center, Beirut, Lebanon; ^3^ Division of Infectious Diseases, Department of Pediatrics and Adolescent Medicine, American University of Beirut Medical Center, Beirut, Lebanon; ^4^ Department of Surgery, American University of Beirut Medical Center, Beirut, Lebanon; ^5^ Division of Pediatric Cardiology, Department of Pediatrics and Adolescent Medicine, American University of Beirut Medical Center, Beirut, Lebanon

**Keywords:** infective endocarditis, streptococcus viridans, congenital heart disease, pediatric population, complex heart disease

## Abstract

**Introduction:**

Infective endocarditis is an infection of the endothelial surfaces of the heart. It is more prevalent in adults but its incidence in the pediatric population has been on the rise. The most important factor remains congenital heart disease and the most isolated pathogen is viridans group streptococcus.

**Methods:**

In this manuscript, we present a 20-year experience of a major referral tertiary care center in diagnosing and treating pediatric patients with endocarditis. A retrospective analysis of records of patients who were diagnosed with infective endocarditis under the age of 18 years is presented in this study. Variables relating to the demographic, imaging, microbiologic and pathologic data are described. Outcomes relating to complications and need for surgical repair are also portrayed.

**Results:**

A total of 70 pediatric patients were diagnosed with endocarditis in this time interval. The medical records of 65 patients were comprehensively reviewed, however the remaining 5 patients had severely missing data. Of the 65 patients, 55.4% were males, and the mean age at diagnosis was 7.12 years. More than half of the population (58.5%) had vegetation evident on echocardiography. The pulmonary valve was the most commonly affected (50%), followed by the mitral valve and tricuspid valves (15.6%). Most patients received empiric treatment with vancomycin and gentamicin. Viridans group streptococcus was the most frequently isolated organism (23.4%).

**Conclusion:**

Among pediatric patients diagnosed with endocarditis in this study, data pertaining to valve involvement and microbiologic information was consistent with the published literature. The incidence of complications and the need for surgical repair are not significantly correlated with demographic and clinical variables.

## 1. Introduction

Bacterial infective endocarditis (IE) is defined as an infection in the endothelial surface of the heart which include the heart's endocardium and its valves. The infection eventually damages the endocardial tissue and valves which might be irreversible. IE has been reported to be more prevalent in adults compared to adolescents and children (1). There exist several risk factors for IE. The most common of which remains the presence of congenital heart disease (CHD). In fact, CHD can increase the risk of contracting IE in children by 10–140 folds when compared to the general population (1). It is even estimated that up to 70% of IE in children is seen in patients with CHD (2). However, CHD has not been associated with increased mortality of IE in children (2, 3). The overall incidence of IE in children and adolescent has been on the rise. With the new novel therapeutics and surgical interventions, complications secondary to post-operative care and cardiac catheterization have increased the likelihood of contracting endocarditis (4, 5). Indeed, infective endocarditis as an entity is rare in both adults and children. The most recent data reports its incidence in children to be around 0.43–0.69 per 100,000 children per year (2). It has been reported that the mortality of pediatric IE ranges between 1% and 5%, but can reach up to 10% (2, 6). The most commonly associated organism with bacterial endocarditis is streptococcus viridans group, followed by Staphylococcus aureus (5). Other organisms known to cause infective endocarditis include coagulase-negative Staphylococci, Streptococcus pneumonia, Enterococcus species, HACEK, candida, and aspergillus (7). The literature regarding the incidence of endocarditis in the pediatric population in the Middle East region is lacking. In addition, the association between the incidence of endocarditis and its complications with the presence of CHD along with other clinical and demographic characteristics is not properly documented. In this manuscript, we describe a 20-year experience of the American University of Beirut Medical Center (AUBMC), a tertiary referral center in Lebanon, in managing endocarditis in the pediatric population. This center is a leading referral center in Lebanon and the Middle East. We examine the patients’ demographic characteristics, the microbiologic profile, the use of antimicrobials, the presence of complications, and mortality. In addition, we try to examine possible risk factors for early surgery and complications.

## 2. Methodology

### 2.1. Study design

This is a retrospective chart review study, conducted at the American University of Beirut Medical Center. Data was collected from the medical records of patients presenting to the Children's Heart Center at the American University of Beirut Medical Center between January 2000 and December 2020, inclusive. The collected data included information pertaining to the patients' demographic characteristics, medical history, laboratory results, imaging findings, procedures, surgical details, progress notes, and the prescribed medications. Patients who had a final diagnosis of infective endocarditis and who were 18 years of age or below at the time of diagnosis were included in the study. The study excluded patients who were above 18 years of age at the time of diagnosis, and those who didn't have a confirmed diagnosis of endocarditis. The diagnosis of bacterial infective endocarditis was performed according to the Duke Criteria reported by the Center for Disease Control and Prevention (8). Each medical chart was initially reviewed by a single investigator. Another investigator independently reviewed the charts which was reported to have missing information, in order to make sure that as much data as possible was collected. Whenever any uncertainty is present, the investigator would review and resolve the issue with the principal investigator. The study was approved by the institutional review board at the AUBMC under the ID: BIO-20190187.

### 2.2. Definitions

The collected demographic information included patients' age, gender and geographical area of residence. For the purpose of this study, the official national classification which sorts the country into eight different areas (Akkar, Baalbek-Hermel, Beirut, Beqaa, Mount Lebanon, Nabatieh, North and South Lebanon) was used. The collected laboratory information included the results of the blood culture, tissue culture, pathology result, and the 16 s ribosomal RNA. In general, a blood culture is initially obtained in all patients who are suspected of having infective endocarditis. When there remains a high clinical suspicion of endocarditis in the setting of repeated negative blood cultures, a 16 s ribosomal RNA might be obtained. This test allows for the identification of residual bacterial RNA present in the blood in patients who fail to grow any bacterial colonies especially in the setting of broad-spectrum antibiotics use. Pathological examination of the affected cardiac areas remains the test with the highest specificity to diagnose endocarditis but is not often performed as such invasive procedure presents its own challenges through cost and practicability. The pathology results were reviewed for all patients who had a surgical intervention and during which a pathology sample was obtained. Furthermore, reviewed images included x-rays, echocardiography, CT scan, and MRI results. The echocardiography was performed in all patients included in this study. It was considered supportive for the diagnosis of endocarditis if it reveals one of the following: new vegetation, abscess formation, or dehiscence of an old prosthetic valve. For patients with suspected endocarditis and unremarkable echocardiography, a computed tomography (CT) scan of the chest was performed to evaluate for mediastinitis or abscess formation.

### 2.3. Statistical analysis

All analysis was conducted and represented using SPSS and Microsoft Excel. Continuous variables including age were represented as mean ± standard deviation. Categorical variables, including gender, geographic area, CHD complexity, culture results, 16 s results, type of valvular endocarditis, type of prosthetic endocarditis, affected valve, valve surgery, and complications are presented as frequency or percentage. To test the association between independent variables and primary outcomes, a *χ*^2^ test were used. Fisher exact modification was used if any cell contained a variable less than 5. Any *P*-value less than or equal to 0.05 was considered significant. A *P*-value less than 0.05 was considered significant.

## 3. Results

During the 20-year study period, 70 patients were diagnosed with infective bacterial endocarditis and were 18 years or below at the time of diagnosis. The medical charts of 65 patients were comprehensively reviewed, relevant data was collected and included in the analysis. However, the medical charts of five patients were found to be severely missing information, therefore these patients were not included. Males constituted 55.4% of the population. The mean age at diagnosis was 7.12 ± 5.0 years. The youngest patient was 2 months old while the eldest was 17 years old. Patients were coming from different geographic areas. Mount Lebanon had the highest number of patients (41.5% of our population), followed by Beirut and North Lebanon, each contributed to 13.8% of the patients. Three patients were from Syria, while only one patient was from Iraq.

**Figure 1:**
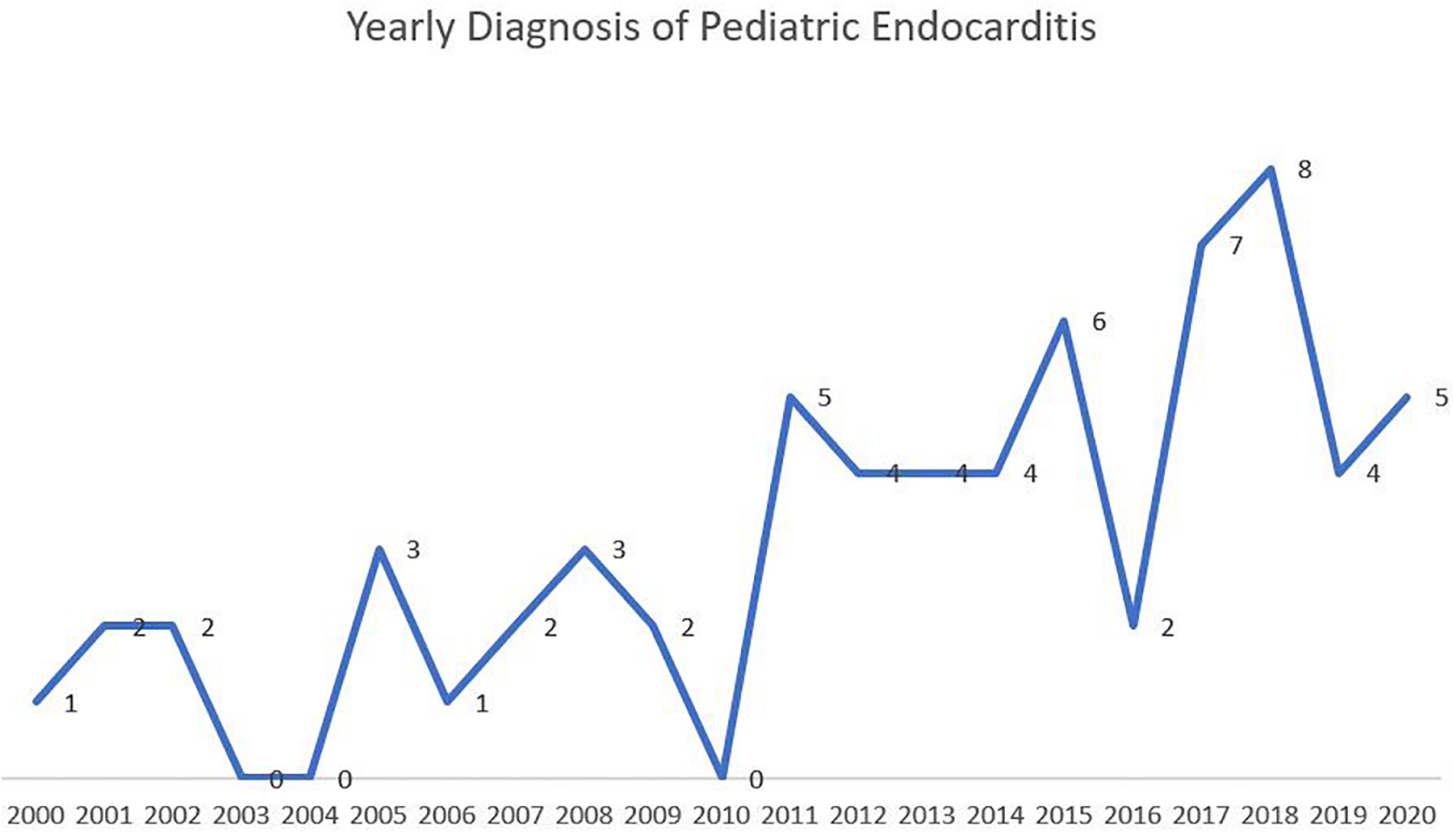
The yearly distribution of pediatric infective endocarditis.

Indeed, the presentation of patients has greatly varied. A proportion of patients presented to the outpatient clinic with history of prolonged fever with or without associated symptoms. They were found to have vegetation on imaging, and were subsequently referred to the emergency room. Others didn't present with vegetation, but had high suspicion of endocarditis, therefore were referred to the emergency room for evaluation. This subacute presentation of IE was quite prevalent. A significant group of patients presented directly to the emergency room for fever that is associated most of the time with non-specific symptoms of fatigue, hypoactivity and lethargy. Luckily very small proportion of patients presented with a picture of fulminant endocarditis leading to hemodynamic instability. Figure 1 shows the yearly distribution of endocarditis. A progressive increase in the number of yearly pediatric cases is noted.

All but nine patients (13.8%) had cardiac disease. VSD was the most common congenital heart defect accounting for 20%, followed by Tetralogy of Fallot accounting for 10.8% of the patients. Table 1 represents the distribution of patients by heart anomaly. For the diagnosis of endocarditis, blood cultures were obtained from all patients before initiating antibiotics. Of the study population, 38 patients had their blood cultures positive for at least one organism (58.5%), while the remaining 41.5% failed to grow any organism on repeated blood cultures. Of those patients, only eight (12.3%) had 16 s ribosomal RNA testing. It came back positive in half of the patients (4/8 patients). Tissue culture was only performed in 10 patients (15.3%), and yielded positive result in eight patients (80% of those who had tissue culture examination).

**Table 1:** Distribution of patients by type of congenital heart lesion.

Diagnosis	Frequency	Percentage (%)
Anomalous left coronary artery	2	3.1
AVC	4	6.2
COA	1	1.5
DORV	4	6.2
MR	4	6.2
MS	2	3.1
Normal heart structure	9	13.8
PS	4	6.2
Pulmonary atresia/hypoplasia	3	4.6
RVOT obstruction + VSD	1	1.5
Supravalvular AS	1	1.5
TGA	6	9.2
TOF	7	10.8
Truncus arteriosus	3	4.6
Valvular AS	1	1.5
VSD	13	20

PS, pulmonary stenosis; DORV, double outlet right ventricle; VSD, ventricular septal defect; CoA, Coarctation of the aorta; AS, aortic stenosis; AVC, atrioventricular canal; TGA, transposition of great arteritis; MR, mitral regurgitation; MS, mitral stenosis; TOF, tetralogy of fallot; RVOT, right ventricular outflow tract.

Interestingly, more than half of the population (58.5%) had vegetation evident on echocardiography. The pulmonary valve was the most commonly affected (50% of the documented affected valves), followed by the mitral valve and tricuspid valves (15.6%). Three patients had double valve involvement: mitral and aortic valves. The pacemaker was also affected in three patients. A total of 21 patients required early or late surgical intervention, being for valvar repair, abscess drainage or conduit change. This was unequally distributed between 16 patients undergoing early surgery and five patients undergoing late surgery. The number of patients who have suffered complications from endocarditis remains considerably moderate, representing 23% of the population. The complications varied between: atrioventricular block, secondary sepsis, embolism, and cardiogenic shock, severe pericardial effusions, brain abscess and splenic infarcts. Two patients died after being diagnosed with endocarditis (see Table 2).

**Table 2:** Demographic and clinical characteristics of the patients.

	Frequency	Percentage (%)
Gender		
Male	36	55.4%
Age (mean, SD)	7.12	(5.0)
2 months	1	1.5
4 months	1	1.5
9 months	1	1.5
1 year	5	7.7
1.5 years	1	1.5
2 years	3	4.6
3 years	8	12.3
4 years	5	7.7
5 years	4	6.2
6 years	2	3.1
7 years	3	6.2
8 years	7	10.8
9 years	1	1.5
10 years	4	6.2
11 years	1	1.5
12 years	4	6.2
13 years	2	3.1
14 years	4	6.2
15 years	2	3.1
17 years	4	6.2
Location		
Akkar	3	4.6%
Baalbek	4	6.2%
Beirut	9	13.8%
Mount Lebanon	27	41.5%
North Lebanon	9	13.8%
South Lebanon	7	10.8%
Beqaa	2	3.1%
Syria	3	4.6%
Iraq	1	1.5%
Blood culture		
**Negative blood culture**	**27**	**41.5%**
*16 s*	*8*	
Positive	4	50%
Negative	4	50%
*Tissue Culture*	*10*	
Negative	2	20%
Positive	8	80.%
**Positive blood culture**	**38**	**58.5%**
Vegetation on imaging	38	48.5%
Endocarditis		
Native valvular	18	47.4%
Prosthetic	20	52.6%
**Prosthetic Endocarditis**		
Valvular/Conduit	14	70%
Pacemaker	3	15%
Conduit + Patch	2	10%
Patch	1	5%
**Affected Valve**		
Pulmonary	16	50%
Aortic	3	9.3%
Mitral	5	15.6%
Tricuspid	5	15.6%
Two valves	3	9.3%
Patients undergoing Surgery	21	20%
Surgery timing		
Early surgery	16	33.3%
Late surgery	5	16.7%
Type of complications		
Embolic events	2	13.3%
Atrioventricular block	4	26.6%
Secondary Sepsis	2	13.3%
Heart Failure	2	13.3%
Cardiogenic shock	2	13.3%
Neurologic	2	13.3%
Death	2	13.3%

Most patients were treated with a double antibiotic regimen. This constituted vancomycin and gentamycin for most patients (30.7%) followed by vancomycin combined with meropenem (16.9%), or with ceftriaxone (13.9%). This was subsequently de-escalated to a single agent, when possible, based on the anti-microbial sensitivities. Other patients were switched to a single-antibiotics coverage after responding for at least two weeks. Either vancomycin or gentamycin alone were used as a single medication for a longer period. Other frequency used empiric agents include cefepime, meropenem and amikacin. Rifampin was used in four patients. Additionally, two patients required anti-fungal coverage with caspofungin.

**Figure 2:**
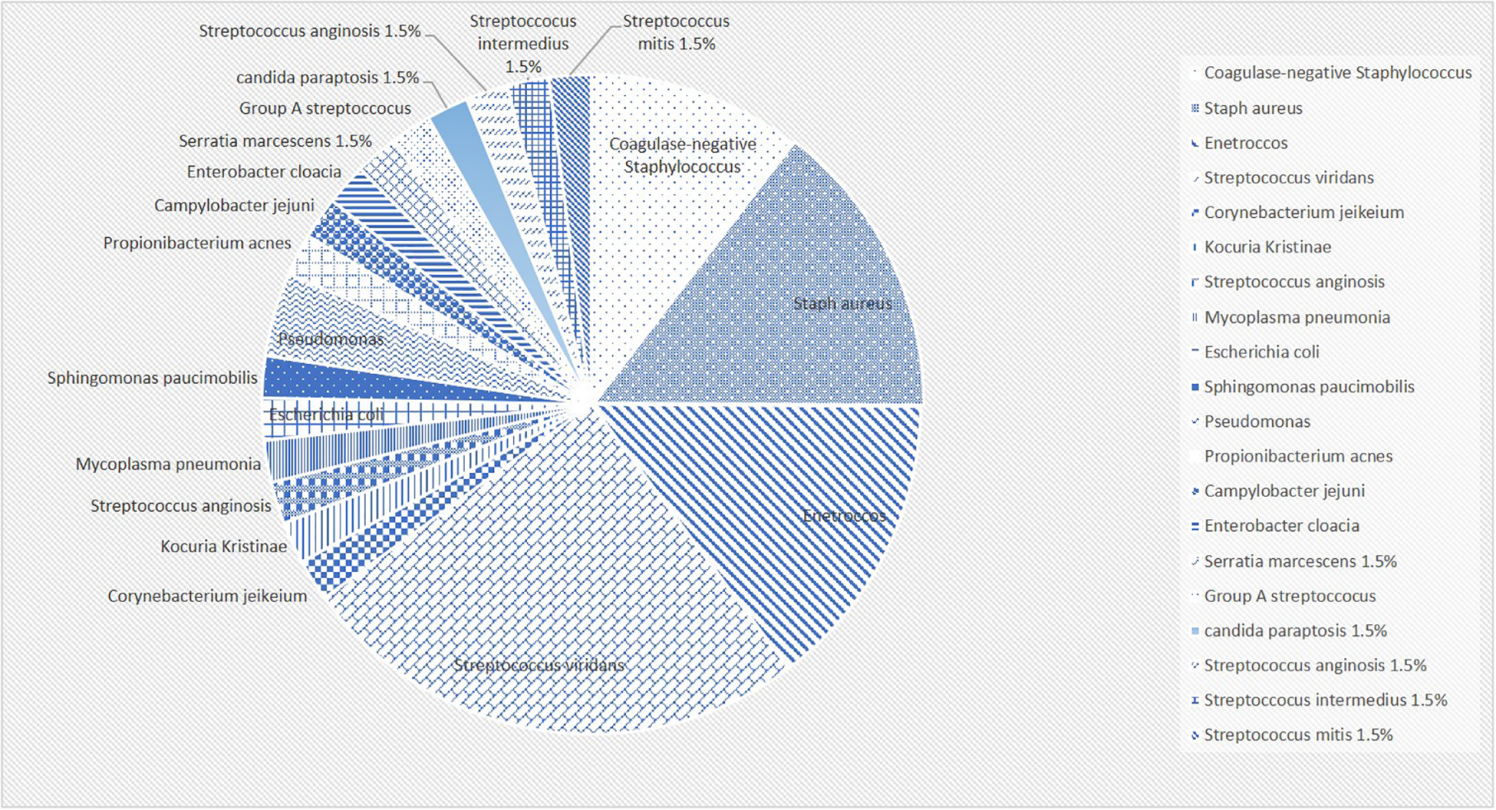
The percentages of detected organisms in a pie chart.

The most frequently recovered causative organism was viridans group streptococcus (18.4%). This was followed by Enterococcus and Staphylococcus aureus (10.7% each). The rest of the organisms that were recovered from the culture are highlight in Figure 2.

Males were significantly more likely to require valvular surgery. No significant difference was noted in terms of incidence of complications and the need for surgery between those with native vs. prosthetic valve endocarditis (*P*-value = 0.736). Similarly, no significant difference was found in terms of association between which valve is affected and the occurrence of early complication or the need for surgery (Table 3).

**Table 3:** Outcomes of early complications and valvular surgery analyzed against the different demographic and clinical characteristics.

Variable	Early complications		*P*-value	Valvular surgery		*P*-value
	Yes (%)	No (%)		Yes (%)	No (%)	
*N*	15 (23)	50 (77)	** **	13 (20)	44 (80)	
Gender			0.85			0.027*
Male	8 (22)	28 (78)		11 (84.6)	25 (48.1)	
Female	7 (24)	22 (76)		2 (15.4)	27 (51.9)	
Blood culture	8 (53.3)	30 (60)	0.767	7 (53.8)	31 (59.6)	0.76
Vegetation abscess	8 (60.0)	30 (53)	0.646	9 (100)	29 (100)	
Native endocarditis	4 (36.4)	14 (43.8)	0.736	4 (50)	14 (40)	0.701
Prosthetic endocarditis			0.691			0.459
Conduit/valve	2 (18.2)	12 (34.3)		4 (44.4)	10 (27)	
Pacemaker	1 (9.1)	2 (5.7)		0 (0)	3 (8.1)	
Patch	0 (0)	1 (2.9)		0 (0)	1 (2.7)	
Conduit + Patch	(9.1)	1 (2.9)		1 (11.1)	1 (2.7)	
Valve affected			0.989			0.113
Tricuspid valve	1 (6.7)	4 (8)		0 (0)	5 (9.6)	
Pulmonary valve	3 (20)	13 (26)		3 (23.1)	13 (25)	
Aortic valve	1 (6.7)	2 (4)		2 (15.4)	1 (1.9)	
Mitral valve	1 (6.7)	4 (8)		1 (7.7)	4 (7.7)	
Two valves	1 (6.7)	2 (4)		2(15.4)	1(1.9)	

* Significant *P*-value.

## 4. Discussion

Infectious endocarditis is defined as a bacterial or fungal infection primarily inflicting damage on the endocardium and heart valves. The exact incidence of IE in the pediatric population is not well documented. Most of the evidence present in the literature reflects single-center or single-country studies. IE has been present for over 50 years and is being controlled with the advent of antibacterial agents. In addition, the microbiology of the infections has been changing with time (9). For instance, between 1952 and1957, streptococcus viridans was the most common pathogen encountered while in the (1958–1963) era, it was Staphylococcus aureus (9).

Although IE has led to significant mortality that varied between 5% and 10%, its incidence in the pediatric population remains less than that of adults (10). The number of patients with IE has been on the rise in the United States with its incidence increasing from 11 cases per 100,000 per year in the year 2000 to 15 cases per 100,000 per year in the year 2011 (10). This might be explained by the increase in the use of prosthetic devices. In addition, there has been an increase in the use of central venous catheters which also carry a high risk of disseminated infection (11). However, no change in the rate of increase was noted around the time of the implementation and modification of the 2007 American Heart Association (AHA) guidelines that limited prophylaxis to high-risk groups (10). More recent data from a multicenter study estimated that there were between 0.05 and 0.12 IE cases per 1,000 hospital admissions during the period spanning 2003–2010 (12). The incidence was also higher in those with underlying CHD. Over the study period which spanned around 11 years, the incidence of IE in children was 0.43 per 100 K, and it was shown to remain stable all throughout that period. Most of the patients were in the age range of 11–19 years (56.2%) (12). The lowest incidence was in patients below 1 year of age. 53.5% of the patients suffered from underlying cardiac conditions and around 7.5% had prosthetic valves, 3.9% had a cardiac device and 3.6% had cardiomyopathy (11).

In this study, 65 pediatric patients diagnosed with endocarditis were described and analyzed. The average age of our population (7.1 years) was relatively lower than that in the literature. Our youngest patient aged 2 months. The yearly distribution of IE has shown a gradual increase over the years (2000–2020). Similar to the global trend, this increase is most likely attributed to the sharp increase in surgical intervention and invasive procedures in patients with CHD. Interestingly, a notable increase in the incidence of IE in our population was noted after 2011. This coincides with the recruitment of a specialized pediatric cardiac surgeon at our institution, therefore with increasing number of yearly cardiac surgeries in the pediatric population.

Although the incidence of IE has been on rise, the microbiologic profile has been consistent. Staphylococcus and streptococcus viridans, both normal oral floras, remain the most common organisms associated with bacterial endocarditis with or without CHD (9).

In addition, it is suggested that a difference exists in the etiology of IE depending on CHD status. For instance, it was found that patients with CHD were more likely to suffer from streptococcus species associated with IE, whereas S. aureus was more common in children without CHD. One mode of pathogenesis of atypical bacteria involves the formation of biofilm which will help attach the bacteria to the endothelial lining (6, 9). A certain trend with organisms was observed between different age groups with Streptococcus species being the most common in older patients. This may be related to Streptococcus being more likely to be a cause of IE after oral procedures, which older patients are more likely to undergo (13).

Different valves can also lead to different rates of IE. For instance, the Melody valves, which have been in increasing use since the year 2000, contain an intrastent bovine jugular vein (BJV). The incidence of infective endocarditis on these valves had been reported to be relatively high varying between 3% and 14% (6). In addition, although the incidence of IE is higher in BJV valves compared to other right ventricle–to–pulmonary artery conduits, it did not differ between valves that were placed surgically and those placed percutaneously (14).

A possible explanation for their higher rates of IE might be due to Melody valves acting as substrates which cultivate an environment for IE. In addition, the BJV valves have inherent asymmetry which lead to damage to endothelium due to turbulent flow. This in turn leads to platelet and fibrin accumulation that provides a good environment for the deposition of bacteria.

Another reason behind the increased incidence of IE in this group might be due to the immunologic reaction to bovine tissue in the body which also predisposes to the formation of non-bacterial thrombotic endocarditis (14). This group of patients might benefit from prophylactic antibiotics as they are at a high risk of developing infections.

In our population, only one patient had melody valve. This patient had double outlet right ventricle with sub-pulmonary VSD. He had multiple previous surgeries that include repair of coarctation of the aorta, pulmonary band placement and Damus procedure. In addition, he had percutaneous insertion of Melody valve in another institution. Three years following the valve placement he developed IE involving the Melody valve, that was successfully treated with a course of antibiotics.

When comparing different valves, it has been shown that the bovine jugular vein valves and contegra valves have a higher incidence of IE compared to their counterparts. This might be explained by the higher incidence of increased bacterial adhesion for S. aureus on BJV valves. Incidence of IE has been shown to be the highest in Contegra conduits (affecting 20.4% of individuals) (6).

In this study, viridans group streptococcus was the most commonly identified organism for IE. Our data goes in line with the literature. Notably, in our study Enterococcus and Staphylococcus aureus shared equal contribution to the positive blood culture (10.7% each). This could be partially explained by the fact that almost half of the patients who had Enterococcal endocarditis had a recent surgical intervention. In addition, a significant portion of our population had culture negative IE (27 patients), which would expose the results to a major bias. However, it remains interesting that two patients had Enterococcal endocarditis but were previously healthy without any underlying cardiac condition.

It was shown that 8%–10% of pediatric patients might develop IE without having any pre-existing structural heart defect or any other known risk factors. In one study, 13.8% of the population didn't have any previous heart lesion. Their infection is usually the result of Staphylococcus aureus bacteremia that inflicts damage upon aortic or mitral valves. Evidently, patients with central lines are at higher risk of developing disseminated infection. Other risk factors associated with IE especially in the adolescents and adult population is the use of intravenous drugs and the presence of degenerative heart disease (15).

The diagnosis of IE can be challenging. In addition, sometimes a definitive diagnosis cannot be made when certain valves are involved, such as the Melody valves. The signs of vegetations might be absent upon initial visualization (6). Transthoracic echocardiography (TTE) is the mainstay of diagnosis with a yield of more than 80% in children. Trans-esophageal echocardiography (TEE) is rarely needed unless there is a high suspicion of aortic root abscess, presence of chest wall deformity, prosthetic valves or other conditions which render TTE non-specific (6). A timely diagnosis is of utmost importance as embolic complications might occur. Intracardiac echo can be employed at times as it can reveal pulmonary valve implantation that is not seen on TEE related infective endocarditis. Another imaging modality that can be used is a CT scan which is important in diagnosing paravalvular complications such as abscess, and it has been incorporated into the new criteria for the diagnosis of IE. Positron-emission tomography (PET-CT) has been growing in importance in cases with suspected IE. It employs uses the fact that infectious and inflammatory foci are metabolically active and have higher uptake of 18F-FDG. Also, adding (18F- FDG) PET–CT as a major Duke criterion has been shown to increase the sensitivity of the modified Duke score from 70% to 97% (8). In our study, PET-CT was performed in four patients, and had positive results in all four cases. However, one patient had recent surgical intervention, which could limit a clear differentiation between infectious and inflammatory condition. However, endocarditis was diagnosed in this case based on a combination of imaging, clinical features and inflammatory markers.

Although several imaging modalities have become available for the diagnosis of IE, blood culture remains the most important factor directing therapy. Blood cultures should be taken as 3 sets, ideally taken 12 h apart, but with the first and last sets being at least 1 h apart. If the patient is unstable, two blood cultures at separate sites should be taken immediately, with a third at least 1 h later and empirical therapy initiated without delay (6). 16 s and 18 s can also be taken as further workup for microbiologic speciation. Prior antibiotic use might lead to culture negative endocarditis. In addition, fastidious microorganisms including Abiotrophia spp., Granulicatella spp, Coxiella spp., Trophyema whipplei, Bartonella spp., Mycoplasma spp., and filamentous fungi should also be considered and added to the differential (11, 16). In the study carried by Gupta et al. in the United States which spanned a period of 11 years, it was found that in 30.2% of the patients, the IE was culture negative (11).

In our population, despite taking several sets of blood culture, a significant percentage of patients (41.5%) continued to have negative results. Thus, identification of causative organism through 16 s RNA becomes essentially useful. Unfortunately, the use of this modality in our study was limited, yet when used, four out of eight patients had positive testing.

Examining tissue culture would be expected to be the most sensitive and specific diagnostic modality, however, its use is severely limited. In this study tissue culture was obtained for 10 patients, and helped to identify the causative agent in 8 patients.

**Figure 3:**
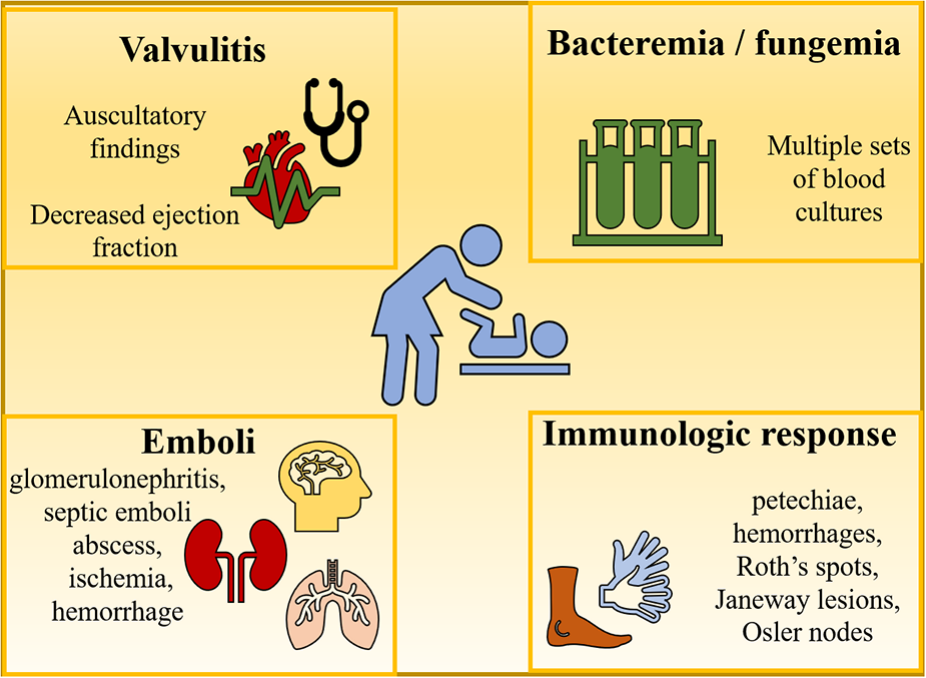
Clinical findings of endocarditis.

Figure 3 depicts the main clinical findings which revolve around 4 phenomena including bacteremia (or fungemia), valvulitis, immunologic responses, and emboli. Valvulitis is responsible for any new auscultatory findings, or it might be the beginning of heart failure. Other extracardiac manifestations including petechiae, hemorrhages, Roth's spots, Janeway lesions, Osler nodes, or splenomegaly are usually more common in adults compared to children (15, 17).

Immunologic problems and embolic phenomena can lead to renal abnormalities (e.g., glomerulonephritis, infarct), symptoms of ischemia and hemorrhage which are the result of emboli lodging in the abdominal viscera, the brain, or the lungs. Rare cases of central nervous system involvement in the form of mycotic aneurysms can occur and lead to catastrophic sequelae (15).

Prevention of infective endocarditis has been implemented early on. The American Heart Association has changed the indications for prophylaxis to become more stringent. The antibiotics used for prophylaxis include amoxicillin and ampicillin (11). Prophylaxis is now recommended for certain subset of patients undergoing dental procedures with certain risk factors including: prosthetic cardiac valve, history of previous episodes of endocarditis, unrepaired cyanotic CHD, postoperative or post interventional catheterization with prosthetic material for 6 months past the intervention date, repaired CHD with residual defect, and post cardiac transplant (11). At the AUBMC-CHC, patients who are included in the recommendations, are educated regarding the use of prophylactic antibiotics. However, real patient's compliance with these recommendations cannot be definitely assessed.

As for treatment, all the patients in our cohort were immediately started on broad spectrum antibiotics after securing blood culture. Most of our cohort were treated with double agents. The most commonly used combination was vancomycin and gentamicin. Ceftriaxone and meropenem were also significantly used. Amikacin was added whenever a patient presented with or developed hemodynamic instability. Two patients required antifungal coverage due to presence of fungemia. After showing good response, antibiotic treatment was de-escalated, and a single agent was used to continue the required treatment duration.

Interestingly, a previous study was conducted at the AUBMC by Bitar et al. that investigates endocarditis in the pediatric population over an 18-year period: between 1977 and 1995 (16). This provides the luxury of comparing the progress of IE in the region. With a simple comparison, one can assess the increase in number of IE cases from 41 pediatric patients within 18 years to 70 patients within 20 years. The percentage of patients who had normal heart structure is comparable (12% vs. 13.8%) between the two studies. Streptococcus viridans and Staphylococcus remained on the top list of causative organisms. The mortality rate had dropped from 29% to 3%, which reflects the advancement in medical techniques. This drop can be attributed to earlier patient's presentation, as well as to the improvement in diagnostic techniques and treatment modalities. For instance, 39% of the patients in the study by Bitar et al., presented in heart failure, compared to only two patients in our study. Additionally, six patients couldn't have echocardiographic assessment due to the unavailability of this technique at the center at that time. Moreover, the use of 16 s RNA was not discussed in the paper, probably due to the lack of this technique at that time. The use of 16 s RNA in our study helped in identifying the causative organism in four patients who had negative blood culture. Therefore, the data extracted from both studies can provide insight into the progression of IE in the region. The incidence of IE is probably going in parallel with the global trend of increasing cases, without significant change in the etiology of the disease. Nevertheless, the mortality and morbidity rates are in decline (17).

## 5. Limitations

Our study has several limitations. The first is inherent to the nature of the study: retrospective chart review, posing potential information and selection bias. Besides, this could limit the assessment of severity of presentation and the course of illness. Another essential limitation is that some medical charts had missing information. This would definitely influence the results, and might prevent the assessment of possible confounding factors. Additionally, this study, although conducted in a tertiary care center and a major referral center, reflects the clinical practice at a single-center in a developing country. It is also limited by the small population size. Larger multi-center studies are essential to better assess the status of endocarditis, its complications and outcomes in the pediatric population in a developing country.

## 6. Conclusion

The prevalence of infective endocarditis has been on the rise in the pediatric population. There is a lack of sufficient registries and data, especially in the Middle East region. This study, with all its limitations, provides an overview regarding different sides of endocarditis in a developing country in the Middle East. Importantly, it sheds the light on the microbiologic nature of IE in Lebanon, the incidence of complications and the need for surgical intervention. It also highlights the medical management of IE in a developing country. Overall, this study should pave the way into further studies that aim to dig deep into the clinical presentation, broader risk factors, and long-term follow up data.

## Data Availability

The raw data supporting the conclusions of this article will be made available by the authors, without undue reservation.
